# An educational intervention to improve hand hygiene compliance in Vietnam

**DOI:** 10.1186/s12879-018-3029-5

**Published:** 2018-03-07

**Authors:** Hang Thi Phan, Hang Thi Thuy Tran, Hanh Thi My Tran, Anh Pham Phuong Dinh, Ha Thanh Ngo, Jenny Theorell-Haglow, Christopher J. Gordon

**Affiliations:** 1 Hung Vuong Hospital, Ho Chi Minh City, Vietnam; 2 Department of Medical Sciences; Respiratory, Allergy and Sleep Research, Uppsala University, Uppsala, Sweden; 3 Sydney Nursing School, The University of Sydney, Sydney, NSW Australia

**Keywords:** Compliance, Developing country, Education, Hand hygiene, Infection

## Abstract

**Background:**

Hand hygiene compliance is the basis of infection control programs. In developing countries models to improve hand hygiene compliance to reduce healthcare acquired infections are required. The aim of this study was to determine hand hygiene compliance following an educational program in an obstetric and gynecological hospital in Vietnam.

**Methods:**

Health care workers from neonatal intensive care, delivery suite and a surgical ward from Hung Vuong Hospital, Ho Chi Minh City, Vietnam undertook a 4-h educational program targeting hand hygiene. Compliance was monitored monthly for six months following the intervention. Hand hygiene knowledge was assessed at baseline and after six months of the study.

**Results:**

There were 7124 opportunities over 370 hand hygiene recording sessions with 1531 opportunities at baseline and 1620 at 6 months following the intervention. Hand hygiene compliance increased significantly from baseline across all sites (43.6% [95% Confidence interval CI: 41.1–46.1] to 63% [95% CI: 60.6–65.3]; *p* < 0.0001). Health care worker hand hygiene compliance increased significantly after intervention (*p* < 0.0001). There were significant improvements in knowledge scores from baseline to 2 months post educational intervention with mean difference standard deviations (SD): 1.5 (2.5); *p* < 0.001).

**Conclusions:**

A simple educational model was implemented in a Vietnamese hospital that revealed good hand hygiene compliance for an extended period of time. Hand hygiene knowledge increased during the intervention. This hand hygiene model could be used in developing countries were resources are limited.

## Background

Hand hygiene (HH) is the primary action to prevent healthcare-acquired infections (HCAIs) and the spread of drug-resistant bacteria. The health burden of healthcare-acquired infections are enormous, with estimates that up to 15% of patients admitted to hospitals in developing countries acquire HCAI, leading to significant mortality rates [1]. This places significant economic burden on health care expenditure, which has been estimated to be approximately €7 billion in Europe [2]. The costs to developing countries health systems is currently unknown but is expected to be a significant economic impact.

Although HH practice is fundamental, maintenance and improvement of the practice is often difficult due to compliance issues with healthcare workers (HCWs) [3]. Hand hygiene compliance rates have been shown to be low in developing countries. Evidence from a large hospital in Vietnam shows an overall HH compliance rate of only 14% compliance, which is well below the minimum standard set by the World Health Organisation (WHO) [4, 5]. Comprehensive guidelines for HH have been developed by the WHO [4], and evidence has shown that interventions such as training, providing hand hygiene facilities, and posting hand hygiene reminders, significantly improved hand hygiene compliance [6–11]. In both developed and developing countries, dramatic increases in hand hygiene compliance have been observed using the WHO guidelines [12–15]. In addition, an extensive literature review found multimodal, implemented HH strategies resulted in higher efficiency than only one intervention in HH behavior change [16].

Improved HH compliance directly impacts on HCAIs rates. Healthcare-acquired infections rates are reduced markedly when HH compliance programs are implemented [11–14, 17–19]. Previously, researchers at a university hospital in Ho Chi Minh City, Vietnam showed HCAIs reductions from 13.1% to 2.1% following a HH program [17]. This intervention used bedside alcohol chlorhexidine hand sanitizers with minimal HCWs training (1 h) to reduce HCAIs incidence in urological patients. Similarly, wound site infection rates in neurosurgical patients (Cho Ray hospital, Ho Chi Minh City, Vietnam) were reduced by 54% in an intervention ward compared to the control ward that reported a 22% increase in infection rates [20]. Whilst, these results are promising, they are limited to single ward comparisons in surgical patients only. As such, the sustainability of infection control practices has not been shown in developing countries. Therefore, the aim of the study was to compare HH compliance rates before and after an educational program for health care workers in a university hospital in Vietnam.

## Methods

This quasi-experimental study examined HH compliance of health care workers in Hung Vuong hospital, Ho Chi Minh City, Vietnam before and after a short-term intensive educational training program. Health care workers from three departments (neonatal intensive care unit (NICU) and nursery, delivery suite, and surgical ward (gynecological surgery and caesarian section)) were monitored for HH compliance for 6 months. Participants completed a baseline questionnaire on HH knowledge and repeated this at 2 months after the intervention. The study included 3 stages: (i) baseline (before the intervention), (ii) short-term intensive educational intervention, and (iii) monitoring of HH compliance during and following 6 months. There were repeated educational training periods and the study was undertaken between August 2014 and May 2015.

Ethical approval was granted by the Institutional Ethical Review Committee of Hung Vuong hospital in July 2014 (158 QD-BVHV). All participants provided written, informed consent prior to commencing the study.

### Participants

Two hundred six health care workers undertook the HH compliance educational program. Participant demographics are shown in Table 1. The study was conducted in three departments and the participants comprised the majority of HCWs staff in these areas: NICU 90%, (72/80 staff), surgical ward 95.3% (82/86), delivery suite 98.1%, (52/53). In addition, anaesthetic technicians participated in this study and their work involved direct patient care. They routinely performed clinical skills such as anaesthetic agent administration and post-operative intravenous therapy management. Their training involved 2.5 years of university education.

**Table 1:** Participant demographics

Characteristics	Data (*n* = 206)
Age, years (SD)	34(8.0) range: 22–54
Female, *n* (%)	177 (85.9%)
Health profession, *n* (%)	206
Doctor	25 (12.1%)
Registered nurse	52 (25.2%)
Midwife	99 (48.0%)
Technician	30 (14.7%)
Experience level, years (SD)	10.0 (6.8) range: 1–34
Clinical setting, *n* (%)	
Delivery suite	49 (23.8%)
Surgical ward	85 (41.2%)
Neonatal ICU	72 (35.0%)
Prior HH training 3 years, *n* (%)	203 (98.5%)
Regular use of alcohol handrub, *n* (%)	203 (98.5%)

### Procedures

#### Baseline

Hand hygiene compliance was monitored for 1 month prior to the education program. This was undertaken using direct observation on the three departments using the WHO Guidelines on Hand Hygiene in Health Care [4]. The HH compliance auditors (six infection control staff trained in direct observation) assessed a total of 1531 opportunities (at least 500 opportunities per department) during the one-month baseline period. Upon completion of the baseline period, HCWs commenced the educational training program. The training was conducted over a two-month period to train the HCWs from the three clinical departments. The six-month HH observation period commenced in each department following the training of HCWs personnel. In this way, the observation period was staggered to ensure the HH observation period immediately followed the training. During each month, for six consecutive months after the educational intervention 200 HH opportunities per department were undertaken. At the end of the study (following 6 months after the intervention), 500 HH opportunities were undertaken in each department in order to match the baseline observation rate. Whilst the health care workers in the three departments were aware of the observation period, with greater than 200 HH observation opportunities accurate, reliable HH compliance data is able to be collected [4]. Hand hygiene opportunities were defined as the moment during health-care activities when HH is necessary to interrupt microorganism transmission by hands and is the denominator for compliance calculations [4]. Hand hygiene compliance is the ratio of the number of performed actions to the number of opportunities.

#### Educational-program

The educational program was developed as a simple HH intervention provided to HCWs over 2 × 3-h sessions. Approximately, 30 participants attended one training program at a time with up to 5 instructors per session. The program consisted of six activities: (i) 10-min video outlining the reasons for hand hygiene, (ii) small group discussion about the reasons for hand hygiene, (iii) a role-playing game where participants had to identify pathogens using an ultraviolet light on participants hands to determine if the hands had been washed, (iv) small group (5–7 participants) discussion to determine the 5 moments of hand hygiene, (v) practice and discussion of procedural aspects of hand washing technique - *six steps of hand hygiene* [4], (vi) lecture about the efficacy of alcohol-based hand-rub compared to water and soap handwashing.

The program was interactive and facilitated discussions were encouraged. Participants were provided with examples and asked to explore conditions when HH was required. In this way, the model used experiential learning of the HCWs and incorporated novel techniques of learning that allowed for consideration of past HH experiences.

### Instruments

Participants completed the HH knowledge questionnaire for HCWs [21]. The questionnaire consisted of 25 items with a combination of yes/no, multiple choice, and true/false formatted questions. These questionnaires were answered anonymously at baseline prior to the educational intervention and at 2 months following the HH observation period of the study. We calculated an aggregate score using similar methods to those described previously [15].

### Data analysis

All data are presented as means and standard deviations (±SD) unless indicated otherwise. All hand-entered data were double-entered and screened for accuracy. Frequencies and percentage statistics were used to describe the demographic variables. Data were assessed for normality using Kolmogorov-Smirnov tests. Data on a whole-group level were found to display a lack of normality (*p* < 0.001), when assessed at a health discipline level, data were normally distributed. To analyse HH compliance over time, a multi-level mixed model analysis was used. We used time (monthly), setting (type of department) and health profession (without interaction term) for fixed effects in the model. Random effects were intercepts as well as random slopes for setting and health profession. Using a two-way factorial ANOVA, pre- and post-knowledge test scores were subjected to a two-way ANOVA with four levels of profession (doctor, nurse, midwife, technician) and three levels of clinical setting (delivery, ward, NICU). All data were assessed for significant interactions using alpha < 0.05.

## Results

During the study we observed documented 7124 opportunities over 370 HH recording sessions. This equated to 132 h and 51 min spent observing HH opportunities. At baseline, we documented 1531 opportunities and 1620 following the educational intervention at 6 months (Table 2). Nurses and midwives were the most common profession monitored and accounted for the majority of HH observations. The proportion of opportunities observed at the three clinical settings were similar before and after the intervention. The most prevalent HH observation was before aseptic task at pre-, and post-intervention (Table 2).

**Table 2:** Hand hygiene opportunities pre-, and post-intervention

Hand hygiene opportunities	Pre-intervention, *n* (%)	Post intervention, *n* (%)
Total opportunities	1531	1620
Times for observation (minutes)	1491	1895
Health profession opportunities		
Doctor	182 (11.9)	142 (8.8)
Nurse/Midwives	1146 (74.8)	1183 (73)
Technicians	199 (13)	295 (18.2)
Clinical setting		
Delivery suite	507 (33.1)	511 (31.5)
Surgical ward	514 (33.6)	564 (34.8)
Neonatal ICU	510 (33.3)	545 (33.6)
Indications		
Before patient contact	327 (21.4)	258 (15.9)
Before aseptic task	482 (31.5)	642 (39.6)
After body fluid exposure risk	357 (27.9)	471 (33.4)
After patient contact	350 (22.9)	298 (18.4)
After contact with patient surroundings	156 (10.2)	143 (8.8)

The WHO guidelines do not distinguish between nurses and midwives and we followed this methodology

### Hand hygiene compliance

**Fig. 1:**
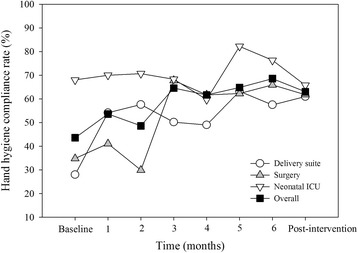
Change in monthly hand hygiene compliance during the study for each hospital department. Error bars have been removed to improve clarity. Commencement of each six-month observation period across the three departments was staggered to align immediately following each department’s HH educational training period

There was the significant improvement of HH compliance following the intervention increasing from 43.6% (95% Confidence interval [CI]: 41.1–46.1) to 63% (95%CI: 60.6–65.3) (*p* < 0.001; Fig. 1). These increases were larger in the delivery suite and surgical ward compared to the NICU which had notably higher HH compliance rates at the commencement of the study. The results from the mixed model showed a significant increase of hand hygiene compliance over time after adjustment for unit and profession (β = 2.69, *p* = 0.03).

### Knowledge-test

The questionnaires were distributed to all 206 participants prior to the education training day and 198 participants completed both the pre-, and post intervention questionnaire (response rate 96.1%). All participants significantly improved knowledge scores from baseline to two-month post-educational intervention (Table 3; all *p* < 0.05). Pre-intervention, technician’s scores were significantly lower than midwives (*p* = 0.018) and registered nurses (*p* = 0.013) [ANOVA Tukey’s post-hoc]. There were no disciplinary differences in post-intervention tests scores (all *p* > 0.05). There were significant interactions between profession and pre-scores (*p* = 0.047) but no effect of setting (*p* = 0.357) or two-way interaction (*p* = 0.694). Similarly, with post-scores, there was a main effect of profession (*p* = 0.046) but not for setting or the interaction.

**Table 3:** Comparison of pre-, and post-intervention hand hygiene knowledge scores

Profession	Pre-score mean (SD)	Post-score mean (SD)	Difference mean (SD)	95% CI	*t*	*p* value
Doctor (*n* = 25)	16.2 (2.8)	18.1 (2.1)	1.9 (2.5)	0.8 to 2.9	3.66	0.001
Registered nurse (*n* = 50)	17.5 (2.2)	18.6 (2.1)	1.1 (2.2)	0.5 to 1.7	3.63	0.001
Midwife (*n* = 95)	17.3 (2.4)	18.8 (2.3)	1.5 (2.5)	1.0 to 2.0	5.90	< 0.001
Technician (*n* = 28)	15.7 (2.9)	17.0 (1.8)	2.0 (3.3)	0.8 to 3.3	3.27	0.003
Total (*n* = 198)	17.0 (2.6)	18.5 (2.2)	1.5 (2.5)	1.9 to 1.2	8.43	< 0.001

## Discussion

The main finding of this study was that HH compliance rates improved significantly and were sustained over a six-month period following the intervention. The increase was more dramatic in the delivery suite and surgical ward compared with the NICU, which had higher baseline HH compliance rates. This study used a more intensive compliance monitoring than previous studies [15, 17], and demonstrated improvement in HH compliance for 6 months. We determined that the educational-based intervention successfully increased HH compliance amongst all HCWs in high turnover clinical departments that are at high risk of HCAIs. Whilst there was an improvement in knowledge scores at the 2 month of the intervention, it was not known if HH knowledge attainment persisted for the entire 6-month study.

Data from our study shows that the HH compliance rates prior to the intervention were low, similar to previous research from Vietnam and other developing countries [5, 20, 22]. Interestingly, in our study we compared three different clinical settings, and found at baseline HH compliance rates were greater in NICU compared to the delivery suite and surgical ward. Studies from industrialised countries have shown that NICU’s have superior HH compliance compared to other hospital settings, especially adult hospital [23, 24] Therefore, these findings are not unexpected. Despite a slight decline in HH compliance in the NICU at 4 months, the level was not lower at the study conclusion. The *Five Moments of Hand Hygiene* [4] had been introduced in the study hospital prior to the study commencement and this may have had a greater influence in the NICU clinical setting; however, the discrepancy between baseline HH compliance rates did not affect the overall improvement rate of HH compliance. The HH compliance improvement in the other departments demonstrated that improvements can be sustained over an extended period of time.

There was a modest degree of variability across the monthly HH compliance scores in the three clinical settings (Fig. 1). The delivery suite recorded the largest change scores pre-, to post-intervention, with a delayed increase in HH compliance after 2 months of the intervention in the surgical ward. It appears that there was no particular pattern to increase for each department, with a late increase in compliance in the NICU that drifted back to baseline levels by the post-intervention stage. However, when averaged across the three clinical settings, there was a steady increase in compliance over the entire six-month period. These data show that increases in repeated measures of HH compliance are not uniform but sustained.

Knowledge improvement following HH educational interventions usually produce positive results in developing countries [25, 26]. However, many pre-, post-study designs measure knowledge close to, or immediately following an intervention. We deliberately chose a follow-up of 2 months to ascertain if knowledge gain was sustained. Whilst the change was statistically significant, there may have been some decay in knowledge as the scores were only marginally above the pre-intervention scores (Table 3). The pre-, and post-test knowledge scores remained low compared to previous data [27]. Nevertheless, the improvement equated to an effect size of 0.62 (Cohen’s D) suggesting that the improvement in HH knowledge was clinically significant [28]. As knowledge change scores improved for amongst all health professions it is possible that this had a positive effect on continuing HH compliance improvement over the 6 months of the study.

Historically, the study hospital used didactic-type educational programs to train HCWs in HH compliance. This was performed yearly and consisted of a two-hour lecture. HH compliance remained low despite these efforts (52%). The current study’s educational program was developed in consultation with HH experts and used WHO training guidelines to frame the evidence-based support of training. Educational programs are effective at reducing HCAIs [29, 30] and these results demonstrate that focused HH training, which incorporates experiential learning, does improve HH compliance for a sustained period.

### Limitations

Although the study was performed in different wards and professions and also used a more intensive compliance monitoring compared with previous studies there are limitations that need to be considered. First, we were not able to systematically measure HCAIs during the study. Whilst there were significant improvements in HH compliance we cannot be confident of decreased HH-related infections. Second, we were unable to expand on the number of clinical settings included in the study due to local logistics with staffing and availability for educational training sessions. As such, we did not include control sites to compare HH compliance. Third, we obtained knowledge scores pre-, and post-intervention but were not able to link these data to the HH compliance data due to confidentiality reasons. This precluded attributing knowledge attainment directly to HH practices. Fourth, we cannot exclude the possibility of the Hawthorne effect which may have elevated the reported HH compliance rates.

## Conclusion

This study demonstrated conclusively that a simple educational intervention can significantly improve HH compliance in clinical settings of high patient turnover. The improvement was sustained over 6 months, and overall monthly data revealed a steady increase in compliance across the study period. Educational HH interventions should aim to measure hand hygiene compliance for an extended observation period to determine effectiveness. This hand hygiene model could be used in developing countries were resources are limited.

## Abbreviations

CI: Confidence interval
HCAIs: Healthcare-acquired infections
HCWs: Healthcare workers
HH: Hand hygiene
NICU: Neonatal intensive care unit
SD: Standard deviations
WHO: World Health Organization
